# Cardiothoracic Imaging in China: Opening Up New Horizons

**DOI:** 10.1097/RTI.0000000000000681

**Published:** 2022-10-21

**Authors:** Long Jiang Zhang, Junjie Yang, Zhengyu Jin, Guang Ming Lu

**Affiliations:** * Department of Radiology, Jinling Hospital, Medical School of Nanjing University, Nanjing, Jiangsu Province; † Senior Department of Cardiology, Sixth Medical Center of PLA General Hospital; ‡ Department of Radiology, Peking Union Medical College Hospital, Peking Union Medical College, Chinese Academy of Medical Sciences, Beijing, China

Since the mid-1990s, China has experienced profound economic and social changes, as well as seismic shifts in residents’ living and health conditions. With the acceleration of population aging and urbanization, the impact of chronic disease risk factors on the health of residents has become more and more significant, among which cardiovascular disease has become the leading cause of death in urban and rural residents in China.^1^,^2^ Approximately 4 million people die of cardiovascular disease in China every year.^2^ To fight against these chronic disorders and emerging infectious diseases, scientists and radiologists from China have made many efforts and important contributions in the past 2 decades.^1^,^2^

Driven by the rapid development of computing power, algorithm optimization and data storage capabilities, Chinese cardiothoracic radiology is experiencing its second development peak. Chinese cardiothoracic radiologists have developed and validated many advanced imaging techniques and integrated them into routine clinical use, such as dual-energy computed tomography pulmonary angiography, computed tomography–derived fractional flow reserve (CT-FFR), and both cardiac magnetic resonance and computed tomography myocardial perfusion techniques. These advanced imaging techniques have been demonstrated to have impressive discriminative capabilities, receiving sufficient attention in Chinese medical research.^3^–^7^ In recent years, a large number of articles on artificial intelligence methods to solve imaging tasks have flooded into medical journals, promoting the interdisciplinary integration of radiology, engineering, computer science, and other fields, culminating in the creation of machine learning based pulmonary nodule detection software and machine learning CT-FFR software in China.^4^,^8^ In this special China issue of the Journal, Tang and colleagues’^9^–^11^ review articles briefly describe the past, present, and future of cardiovascular imaging, thoracic imaging, and imaging in cardiac intervention in China, reflecting the progression and achievements of Chinese Cardiothoracic Radiology over the past 2 decades.

Thoracic and cardiovascular imaging is involved in many complex imaging and analysis procedures. To facilitate the dissemination of advanced cardiovascular imaging techniques in a standardized manner across China, Chinese cardiothoracic radiologists are actively involved in the development, promotion, and application of clinical guidelines, expert consensus, and recommendations. In this special issue, we publish the Chinese expert consensus document addressing CT-FFR on behalf of the Chinese Society of Radiology.^12^ Nowadays, Chinese cardiothoracic radiologists are pushing these advanced cardiovascular imaging techniques into clinical routine applications, an additional effort to lay a solid foundation for future research.

When the coronavirus disease 2019 (COVID-19) pandemic swept the world, causing serious losses of life and property, Chinese cardiothoracic radiologists fought in the forefront against this new disease. They rapidly summarized a series of imaging manifestations, dynamic changes of COVID-19 in chest and heart imaging, reported bioimaging markers of COVID-19, used machine learning techniques to automatically, efficiently and accurately detect COVID-19, classified the severity and assessed the prognosis of the disease.^13^–^18^ The infection control measures and experiences of radiology departments were published and disseminated in some international journals such as the *Journal of Thoracic Imaging* and the *Korean Journal of Radiology*.^19^,^20^ These experiences are still playing an important role in combating COVID-19 around the world.

Although still facing many great challenges such as methodological limitations and the lack of a large prospective multicenter study, Chinese Cardiothoracic Radiology has been exploring safer, more cost-effective, and patient-friendly noninvasive modalities to replace invasive methods. Many knowledge gaps still exist and must be bridged. Multidisciplinary cooperation and breakthroughs are imperative to improve the levels of scientific study and clinical transformation, with sustained cooperation being essential to solve future problems. As Chinese Cardiothoracic Radiology matures, we are eager to unite with more clinical physicians and scientists around the world to bring discoveries in cardiothoracic radiology from the laboratory to the larger public arena of clinical daily practice. Chinese Cardiothoracic Radiology is moving forward with an unyielding steadfastness, embracing its most prosperous era. We firmly believe that with our best efforts, the future of Chinese Cardiothoracic Radiology is promising.

## References

[1] Li X Wu C Lu J . Cardiovascular risk factors in China: a nationwide population-based cohort study [published correction appears in *Lancet Public Health* 2021;6:e271]. Lancet Public Health. 2020;5:e672–e681. 33271080 10.1016/S2468-2667(20)30191-2

[2] Du X Patel A Anderson CS . Epidemiology of cardiovascular disease in China and opportunities for improvement: JACC international. Am Coll Cardiol. 2019;73:3135–3147. 10.1016/j.jacc.2019.04.036 31221263

[3] Zhang LJ Zhao YE Wu SY . Pulmonary embolism detection with dual-energy CT: experimental study of dual-source CT in rabbits. Radiology. 2009;252:61–70. 19561250 10.1148/radiol.2521081682

[4] Tang CX Liu CY Lu MJ . CT FFR for ischemia-specific CAD with a new computational fluid dynamics algorithm: a Chinese multicenter study. JACC Cardiovasc Imaging. 2020;13:980–990. 31422138 10.1016/j.jcmg.2019.06.018

[5] Zhang N Yang G Gao Z . Deep learning for diagnosis of chronic myocardial infarction on nonenhanced cardiac cine MRI. Radiology. 2019;291:606–617. 31038407 10.1148/radiol.2019182304

[6] Li Y Yu M Dai X . Detection of hemodynamically significant coronary stenosis: CT myocardial perfusion versus machine learning CT fractional flow reserve. Radiology. 2019;293:305–314. 31549943 10.1148/radiol.2019190098

[7] Leiner T Bogaert J Friedrich MG . SCMR position paper (2020) on clinical indications for cardiovascular magnetic resonance. J Cardiovasc Magn Reson. 2020;22:76. 33161900 10.1186/s12968-020-00682-4 PMC7649060

[8] Lv W Wang Y Zhou C . Development and validation of a clinically applicable deep learning strategy (HONORS) for pulmonary nodule classification at CT: a retrospective multicentre study. Lung Cancer. 2021;155:78–86. 33761380 10.1016/j.lungcan.2021.03.008

[9] Tang CX Zhang LJ Xu L . Cardiovascular imaging in China: yesterday, today and tomorrow. J Thorac Imaging. 2022;37:xxx–xxx. 10.1097/RTI.0000000000000678 36162066

[10] Fan L Yang WJ Liu SY . Thoracic imaging in China: yesterday, today and tomorrow. J Thorac Imaging. 2022;37:xxx–xxx. 10.1097/RTI.0000000000000670 PMC9592175 35980382

[11] Liu ZN Yang JJ Chen YD . Imaging in cardiac intervention—the Chinese approach. J Thorac Imaging. 2022;37:xxx–xxx. 10.1097/RTI.0000000000000680 36162061

[12] Zhang LJ Tang CX Xu PP . Fractional flow reserve derived from coronary CT angiography: an expert consensus document of the Chinese Society of Radiology. J Thorac Imaging. 2022;37:xxx–xxx. 10.1097/RTI.0000000000000679 36162081

[13] Shi H Han X Jiang N . Radiological findings from 81 patients with COVID-19 pneumonia in Wuhan, China: a descriptive study. Lancet Infect Dis. 2020;20:425–434. 32105637 10.1016/S1473-3099(20)30086-4 PMC7159053

[14] Xu Q Zhan X Zhou Z . AI-based analysis of CT images for rapid triage of COVID-19 patients. NPJ Digit Med. 2021;4:75. 33888856 10.1038/s41746-021-00446-z PMC8062628

[15] Zu ZY Jiang MD Xu PP . Coronavirus disease 2019 (COVID-19): a perspective from China. Radiology. 2020;296:E15–E25. 32083985 10.1148/radiol.2020200490 PMC7233368

[16] Xu PP Tian RH Luo S . Risk factors for adverse clinical outcomes with COVID-19 in China: a multicenter, retrospective, observational study. Theranostics. 2020;10:6372–6383. 32483458 10.7150/thno.46833 PMC7255028

[17] Song F Shi N Shan F . Emerging 2019 novel coronavirus (2019-nCoV) pneumonia. Radiology. 2020;297:E346. 33196374 10.1148/radiol.2020209021 PMC8906333

[18] Ni Q Sun ZY Qi L . A deep learning approach to characterize 2019 coronavirus disease (COVID-19) pneumonia in chest CT images. Eur Radiol. 2020;30:6517–6527. 32617690 10.1007/s00330-020-07044-9 PMC7331494

[19] Xiang KY Zu ZY Lu GM . Coronavirus disease 2019 (COVID-19): Chinese radiologists are acting. J Thorac Imaging. 2020;35:234–238. 32366759 10.1097/RTI.0000000000000528

[20] Chen Q Zu ZY Jiang MD . Infection control and management strategy for COVID-19 in the radiology department: focusing on experiences from China. Korean J Radiol. 2020;21:851–858. 32524785 10.3348/kjr.2020.0342 PMC7289699

